# Supplementary material for: Elaborating the Occurrence and Distribution of Per- and Polyfluoroalkyl Substances in Rivers and Sediment around a Typical Aging Landfill in China
Source: Toxics. 2023 Oct 11;11(10):852. doi: 10.3390/toxics11100852 (PMC10611052; doi:10.3390/toxics11100852)
Supplement: Supplementary file 1 [file toxics-11-00852-s001.zip › toxics-2611215-supplementary.pdf]

This Supporting information contains 3 Tables and 6 Figures which were showed in the followings.

Tables

Table S1

Summary of Water PFAS (mg/L) toxicity to aquatic organisms.

Species	PFBA	PFBS	PFOA	PFOS	Length of exposure	Effect
Lemna minor [1]	4760	7899	-	-	7 d	EC50
Neocaridina denticulate [1]	7866	713	-	-	4 d	LC50
Pseudorasbora parva [1]	14942	3272	-	-	4 d	LC50
Carassius auratus [1]	420	120	-	-	48 d	LC50
Harlequin fly [2]	-	-	0.02	100	20 d	NOAEL
Cladoceran [3]	2180	-	17.95	199.51	48 h	LC50
Trout [4]	-	-	2.50		96 h	LC50
Bullfrog larvae [5]	-	-	99.00	1038	96 h	LC50
Haptophyta [6]	-	-	37.5	163.6	72 h	EC50

LC50, concentration that caused mortality in 50% of the sampled population; EC50, concentration that results in an effect in 50% of sample population; NOAEL, No Observed Adverse Effect Level in experimental study.

Table S2

Sampling information

Dates	Water (No.)	Sediment (No.)
	Sampling site 1 - 12	Sampling site 1, 2, 4 - 12
	Samples number: 12*28=336	Samples number: 11*8=88
03, 2014	W1	S1
05, 2014	W2	
07, 2014	W3	S2
08, 2014	W4	
10, 2014	W5	
02, 2015	W6	
04, 2015	W7	S3
06, 2015	W8	
07, 2015	W9	
09, 2015	W10	S4
10, 2015	W11	
11, 2015	W12	
03, 2016	W13	
05, 2016	W14	S5
07, 2016	W15	
09, 2016	W16	
11, 2016	W17	
04, 2017	W18	
05, 2017	W19	
07, 2017	W20	S6
09, 2017	W21	
10, 2017	W22	
11, 2017	W23	

04, 2018	W24	
06, 2018	W25	S7
07, 2018	W26	
08, 2018	W27	
10, 2018	W28	S8

Table S3

The recoveries of PFASs in water and sediment

PFASs	Water samples				Sediment samples			
	Recoveries		RSD		Recoveries		RSD	
	2ppb	20ppb	2ppb	20ppb	2ppb	20ppb	2ppb	20ppb
PFBA	104%	102%	6%	6%	100%	91%	17%	3%
PFPeA	121%	120%	1%	7%	126%	114%	17%	6%
PFHxA	94%	115%	1%	5%	108%	105%	11%	12%
PFHpA	108%	121%	1%	4%	117%	110%	11%	10%
PFOA	98%	114%	4%	6%	159%	105%	43%	16%
PFNA	103%	118%	2%	4%	120%	110%	9%	15%
PFDA	107%	109%	1%	11%	130%	127%	12%	11%
PFDoDA	208%	118%	8%	10%	112%	125%	18%	17%
PFBS	90%	104%	1%	6%	102%	101%	13%	3%
PFHxS	106%	113%	2%	1%	105%	103%	11%	3%
PFOS	112%	100%	6%	4%	118%	117%	5%	3%

Table S4

HQs for the four PFASs in the water and sediment

Sampling site	Water				Sediment			
	PFBA	PFOA	PFBS	PFOS	PFBA	PFOA	PFBS	PFOS
S1	2.13E-07	1.50E-05	5.32E-08	5.29E-03	1.46E-04	7.89E-03	2.80E-05	3.34E-02
S2	4.35E-07	2.44E-05	1.41E-07	6.92E-03	1.11E-04	1.99E-02	3.51E-05	5.34E-02
S3	9.51E-06	1.26E-03	2.78E-06	4.13E-02	-	-	-	
S4	3.47E-06	1.69E-04	5.26E-07	1.58E-02	8.22E-04	**2.53E-01**	1.04E-04	5.67E-02
S5	2.76E-06	8.25E-05	3.84E-07	1.18E-02	8.86E-04	5.72E-02	5.23E-05	6.27E-02
S6	1.60E-06	5.66E-05	3.07E-07	1.19E-02	2.12E-04	2.99E-02	7.92E-05	5.99E-02
S7	1.05E-06	5.14E-05	2.62E-07	6.01E-03	1.01E-04	2.05E-02	4.08E-05	5.24E-02
S8	7.75E-07	4.03E-05	2.40E-07	1.04E-02	8.13E-05	2.36E-02	9.00E-05	5.26E-02
S9	9.80E-07	4.54E-05	2.46E-07	7.77E-03	3.33E-04	5.34E-02	8.50E-05	6.59E-02
S10	6.11E-07	3.28E-05	1.55E-07	6.51E-03	2.35E-04	5.98E-02	5.26E-05	7.05E-02
S11	2.95E-07	2.68E-05	1.23E-07	6.05E-03	3.37E-04	4.82E-02	1.33E-04	6.66E-02
S12	1.99E-07	9.51E-06	3.16E-08	2.85E-03	3.90E-04	3.60E-02	1.52E-05	3.82E-02

Figure

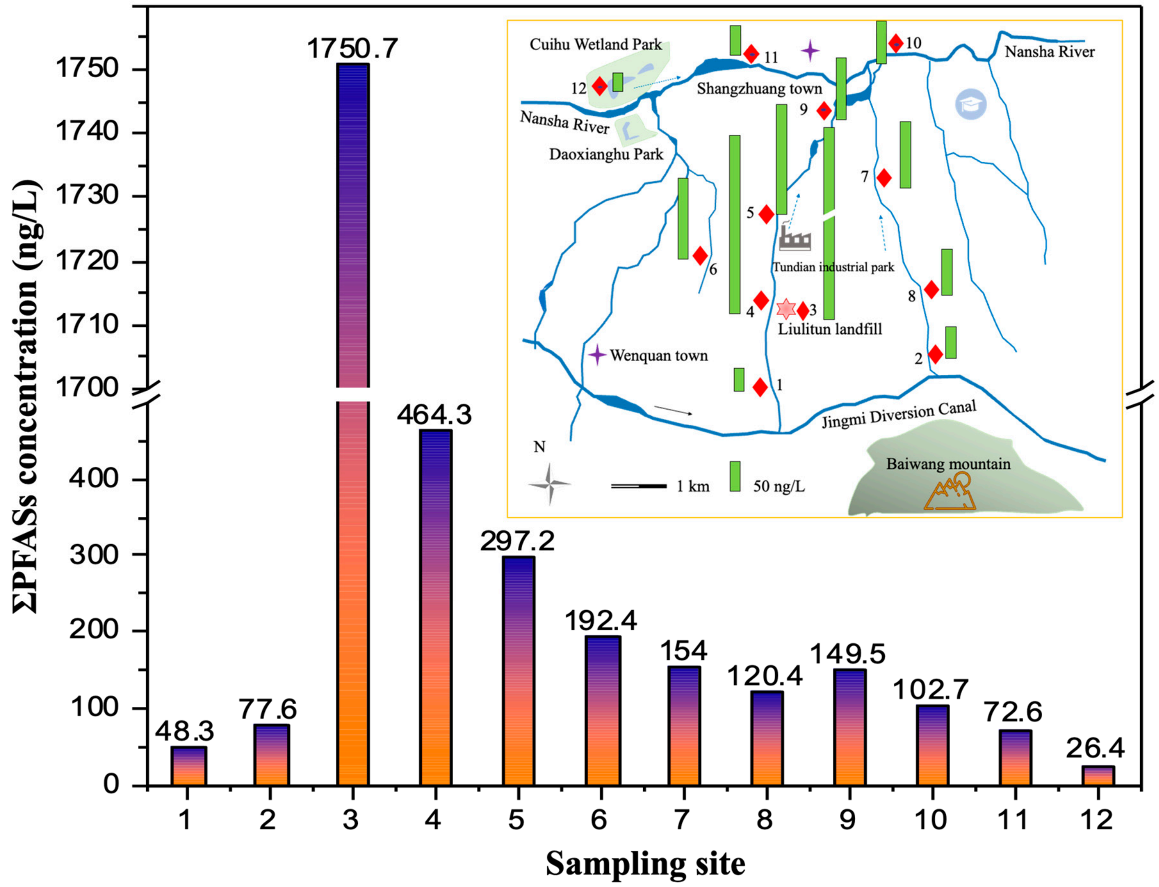

Figure S1 Spatial distribution of average PFASs concentration at each sampling site in water samples in five years

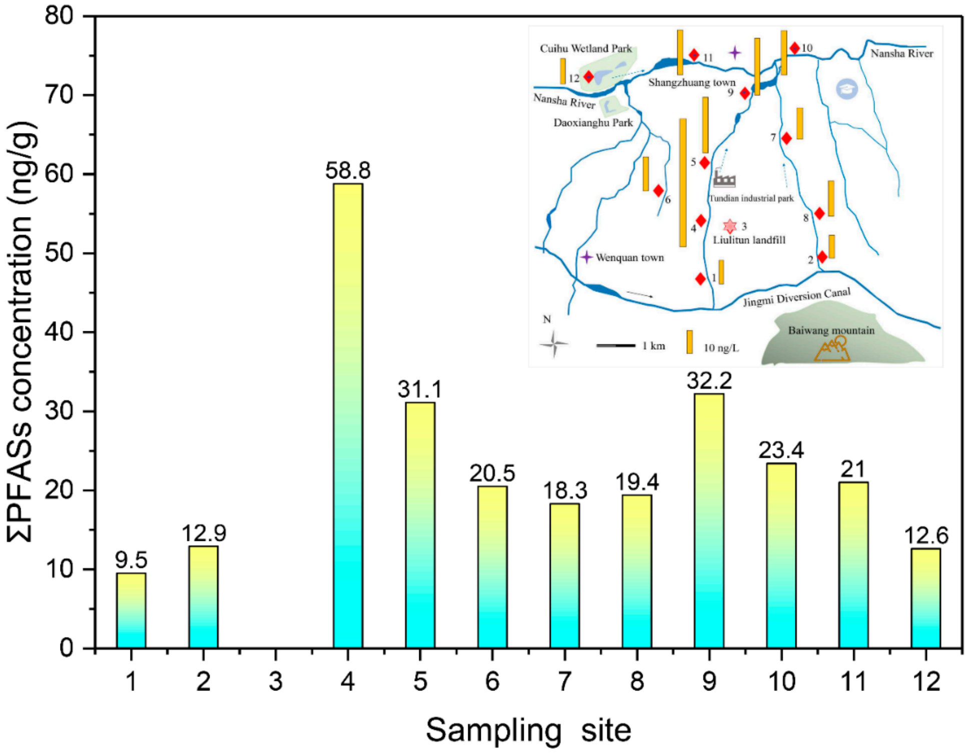

Figure S2 Spatial distribution of average PFASs concentration at each sampling site in sediment samples in five years

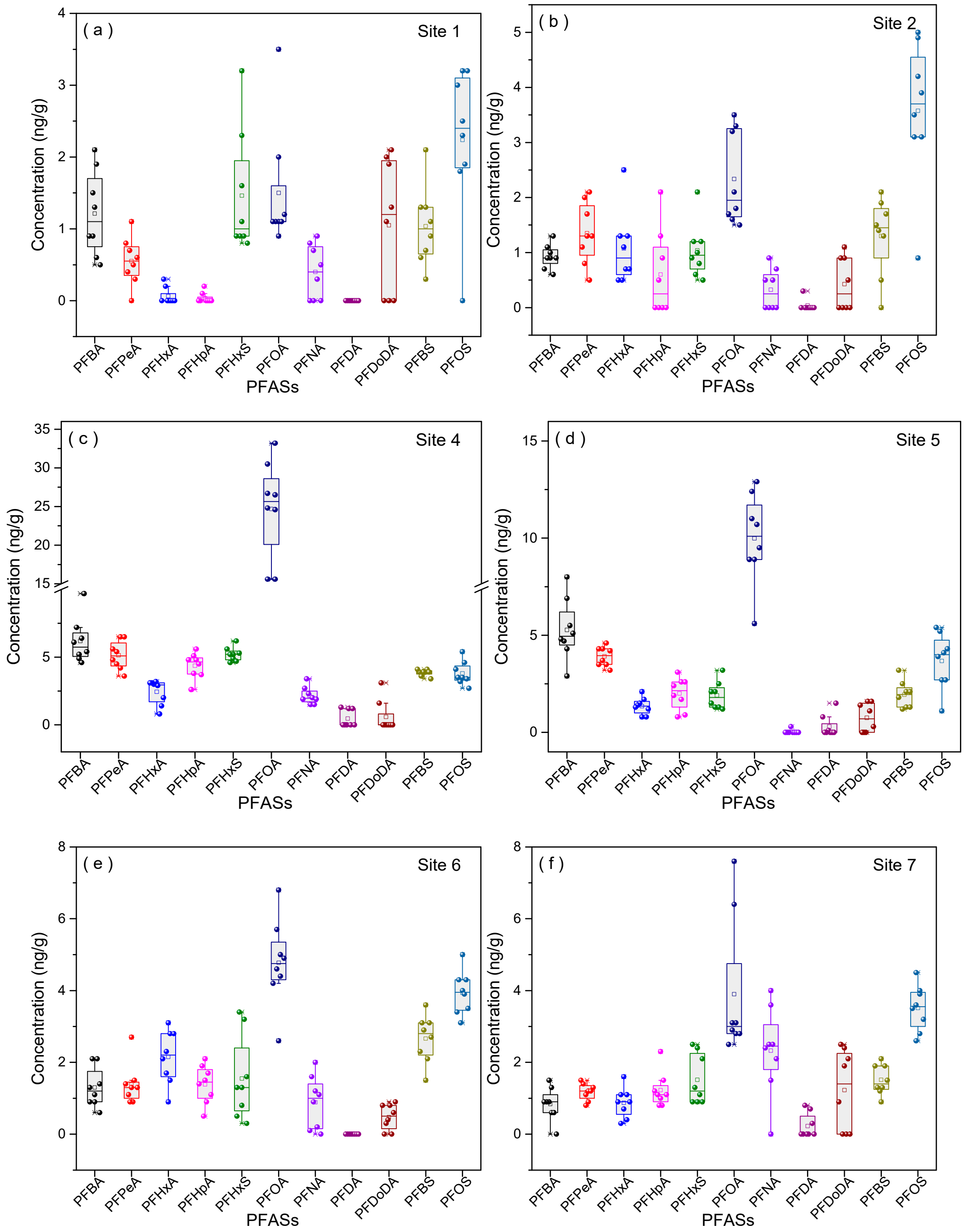

(a)
Site 1
(b)
Site 2
(c)
Site 4
(d)
Site 5
(e)
Site 6
(f)
Site 7
Concentration (ng/g)
PFASs
PFBA
PFPeA
PFHxA
PFHpA
PFHxS
PFOA
PFNA
PFDA
PFDoDA
PFBS
PFOS

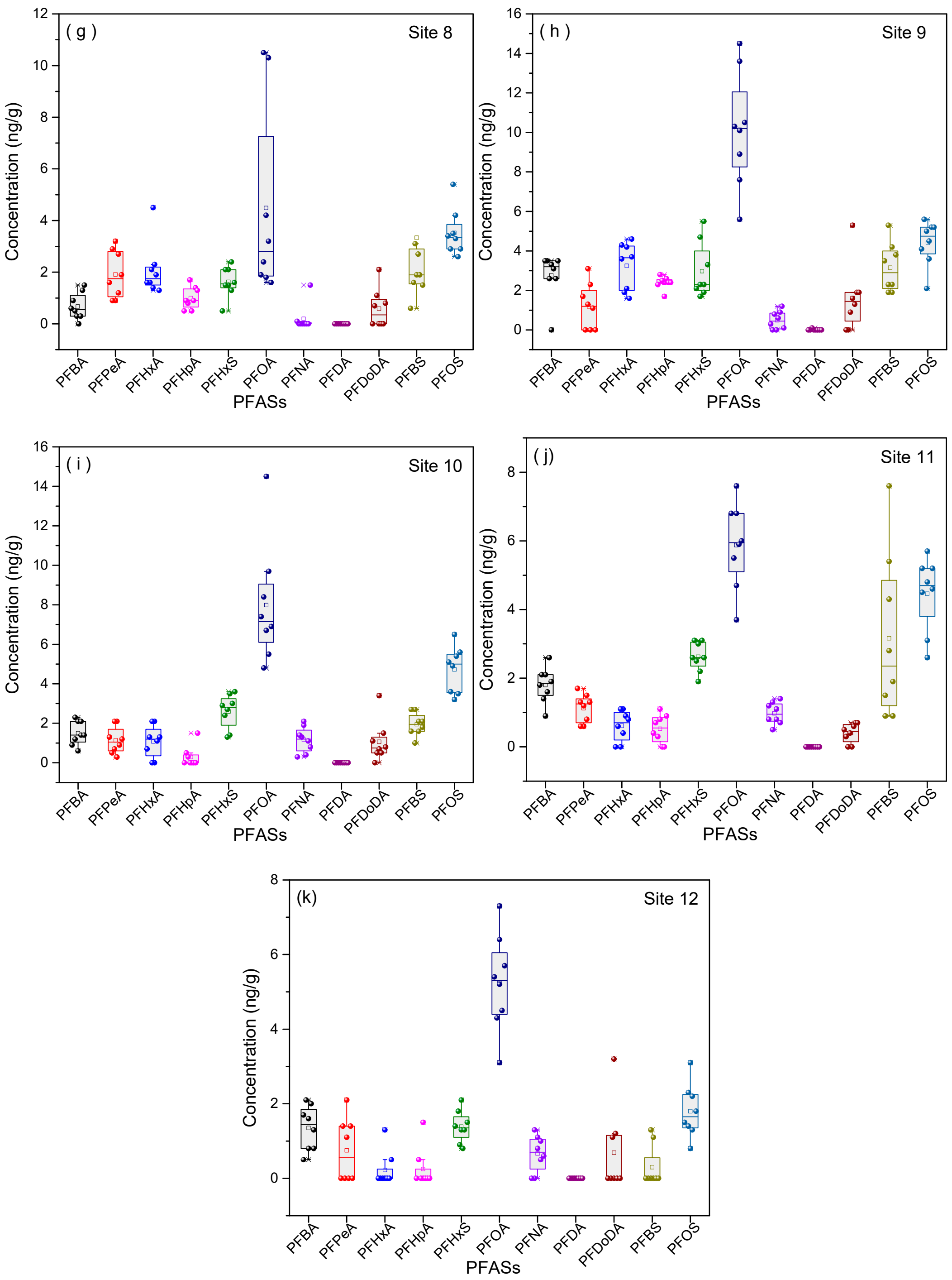

Figure S3 Box-whisker plots of PFASs in sediment from 11 sampling sites around the target landfill in five years. Data with < 80% detected concentrations in all sites were excluded.

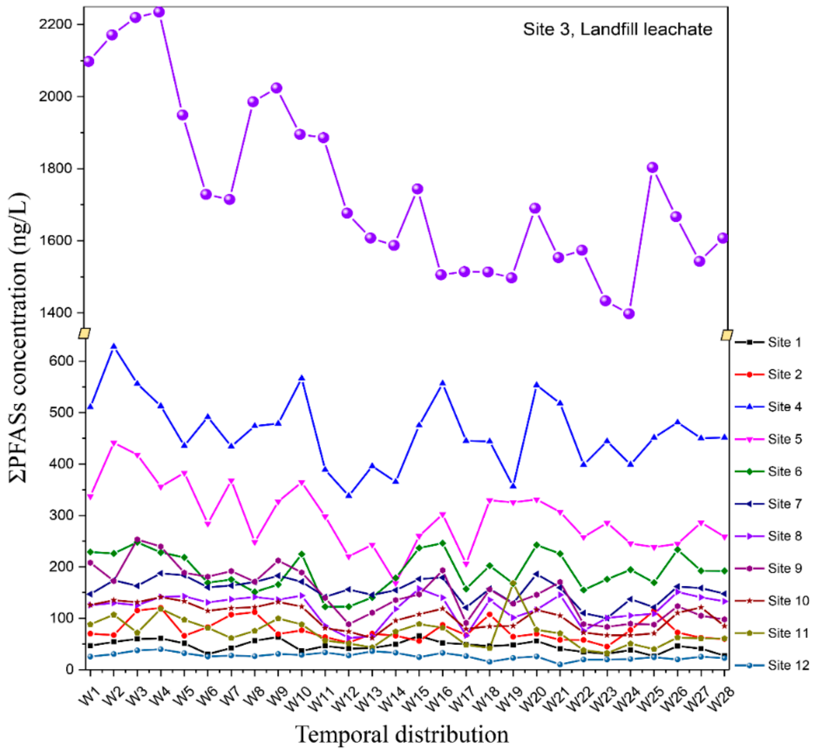

Figure S4 Temporal distribution of average PFASs concentration at each sampling site in water samples in five years

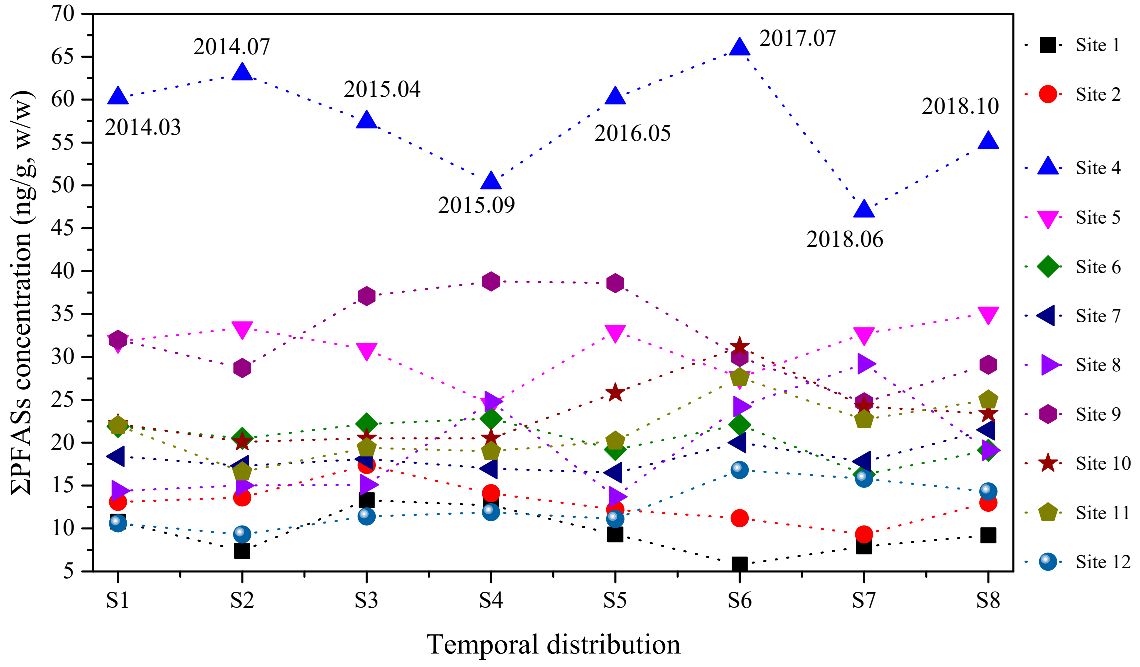

Figure S5 Temporal distribution of average PFASs concentration at each sampling site in sediment samples in five years

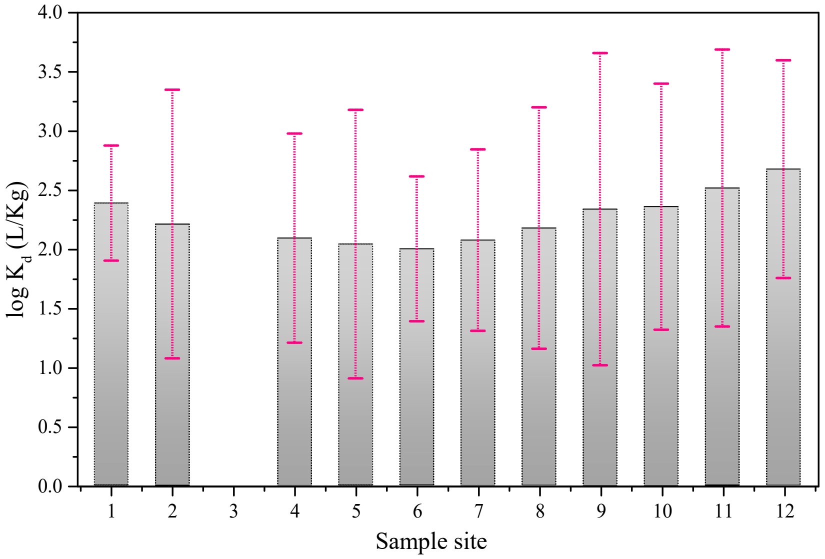

Figure S6 Average logK_d values of PFASs in sediment and river (stream) at different sampling site

References

1. Zhang, Y.-H.; Ding, T.-T.; Huang, Z.-Y.; Liang, H.-Y.; Du, S.-L.; Zhang, J.; Li, H.-X. Environmental exposure and ecological risk of perfluorinated substances (PFASs) in the Shaying River Basin, China. *Chemosphere* **2023**, *339*, 139537-139537, doi:10.1016/j.chemosphere.2023.139537.

2. Marziali, L.; Federica R.; Valsecchi, S.; Polesello, S.; Stefani, F. Effects of perfluoralkyl substances on a multigenerational scale: a case study with Chironomus riparius (Diptera, chironomidae). *Environ. Toxicol. Chem* **2019**, 38, 988e999, doi.org/10.1002/etc.4392.

3. Ji, K.H.; Kim, Y.-H.; Oh, S.; Ahn, B.-W.; Jo, H.-Y.; Choi, K.-H. Toxicity of perfluorooctane sulfonic acid and perfluorooctanoic acid on freshwater macroinvertebrates (Daphnia magna and Moina macrocopa) and fish (Oryzias latipes). *Environ. Toxicol. Chem* **2008**, 27 (10), 2159. https://doi.org/10.1897/07-523.1.

4. Sharpe, Rainie L.; Benskin, Jonathan P.; Laarman, Anne H.; MacLeod, Sherri L.; Martin, Jonathan W.; Wong, Charles S.; Goss, Greg G. Perfluorooctane sulfonate toxicity, isomer-specific accumulation, and maternal transfer in zebrafish (Danio rerio) and rainbow trout (Oncorhynchus mykiss). *Environ. Toxicol. Chem* **2010**, 29, 1957e1966. https://doi.org/10.1002/etc.257.

5. Flynn, R. Wesley, Chislock, Michael F., Gannon, Megan E., Bauer, Stephanie J., Tornabene, Brian J., Hoverman, Jason T., Sepúlveda, Maria S. Acute and chronic effects of perfluoroalkyl substance mixtures on larval American bullfrogs (Rana catesbeiana). *Chemosphere* **2019**, 236, 124350. https:// doi.org/10.1016/j.chemosphere.2019.124350.

6. Mhadhbi, Lazhar, Rial, Diego, Pérez, Sara, Beiras, Ricardo. Ecological risk assessment of perfluorooctanoic acid (PFOA) and perfluorooctanesulfonic acid (PFOS) in marine environment using isochrysis galbana, Paracentrotus lividus, siriella armata and psetta maxima. *J. Environ. Monit.* **2012**, 14, 1375e1382. https://doi.org/10.1039/c2em30037k.
